# Hemoglobin variants shape the distribution of malaria parasites in human populations and their transmission potential

**DOI:** 10.1038/s41598-017-14627-y

**Published:** 2017-10-27

**Authors:** Bronner P. Gonçalves, Issaka Sagara, Mamadou Coulibaly, Yimin Wu, Mahamadoun H. Assadou, Agnes Guindo, Ruth D. Ellis, Mahamadou Diakite, Erin Gabriel, D. Rebecca Prevots, Ogobara K. Doumbo, Patrick E. Duffy

**Affiliations:** 1 Laboratory of Malaria Immunology and Vaccinology, National Institute of Allergy and Infectious Diseases, NIH, Rockville, MD United States of America; 2 Laboratory of Clinical Infectious Diseases – Epidemiology Unit, National Institute of Allergy and Infectious Diseases, NIH, Bethesda, MD United States of America; 3 Malaria Research and Training Center, University of Sciences, Techniques and Technologies of Bamako, Bamako, Mali; 4 Biostatistics Research Branch, National Institute of Allergy and Infectious Diseases, NIH, Rockville, MD United States of America; 5 Present Address: Department of Immunology and Infection, London School of Hygiene & Tropical Medicine, London, UK; 6 Present Address: PATH-Malaria Vaccine Initiative, Washington DC, USA; 7 Present Address: Biologics Consulting Group Inc, Alexandria, USA

## Abstract

Hemoglobin variants C and S protect against severe malaria but their influence on parameters not directly linked to disease severity such as gametocyte carriage and infection chronicity is less well understood. To assess whether these infection-related phenotypes depend on the host hemoglobin genotype, we followed 500 Malian individuals over 1–2 years and determined their parasitological status during monthly visits and incidental clinical episodes. While adults heterozygous for hemoglobin S mutation were less often parasitemic compared to AA adults (odds ratio [OR] 0.50 95% confidence interval [CI] 0.31–0.79, P = 0.003), schoolchildren (but not toddlers or adults) with AC genotype carried parasites, including gametocytes, more often than their AA counterparts (OR 3.01 95% CI 1.38–6.57, P = 0.006). AC children were also likelier to be parasite-positive during the dry season, suggesting longer infections, and were more infectious in mosquito skin feeding assays than AA children. Notably, AC school-aged children, who comprise ~5% of the population, harbor a third of infections with patent gametocytes between May and August, when transmission transitions from very low to intense. These findings indicate that schoolchildren with hemoglobin C mutation might contribute disproportionately to the seasonal malaria resurgence in parts of West Africa where the HbC variant is common.

## Introduction

*Plasmodium falciparum* infections can have diverse clinical presentations, even within the same person over time^1^, and this might impact the potential for onward transmission of the parasite. For example, individuals with asymptomatic infection can carry chronic parasitemia without seeking treatment and might remain infectious to mosquitoes for long periods of time. Consequently, identifying and addressing factors involved in frequent or long-lasting parasite carriage can be important to malaria elimination and control strategies that aim to reduce human-to-mosquito transmission. Here, we assessed whether two hemoglobinopathies, hemoglobin (Hb) S and HbC traits (AS and AC genotypes, respectively), that are frequent in sub-Saharan Africa^2,3^ can influence parasite carriage and transmission.

These two hemoglobin variants have been associated with protection against severe malaria syndromes in numerous epidemiological studies^4^, and mechanistic hypotheses proposed to explain this protection include impaired blood stage parasite development^5–7^, reduced cytoadherence of infected red blood cells^8,9^, which is linked to the redox imbalance of hemoglobinopathies and its effects on the export of parasite-encoded proteins^10,11^, tolerance to malaria infection related to heme catabolism by heme oxygenase-1^12^, or accelerated acquisition of immunity^13–15^. Life-threatening episodes, however, represent only a small proportion of falciparum infections^16^, including in susceptible individuals^1^, and these same mechanisms might regulate other epidemiologically important parameters in non-severe infections, such as duration of parasite carriage and infectiousness. To determine how these variants shape the reservoir of malaria parasites in human populations, we analyzed a longitudinal study with frequent follow-up in an area with seasonal transmission in Mali.

## Methods

### Study population and design

In June 2011, 250 individuals (aged less than 5 [N = 102], between 5 and 15 [N = 73] and more than 15 [N = 75] years) were enrolled, without knowledge of their genetic backgrounds, in an epidemiological study in the village of Bancoumana, southwestern Mali (Supplementary Information Figure S1), in preparation for a Phase 1 malaria vaccine trial. Compounds, which consist of clustered households where individuals of the same family live, were randomly selected using the census list. The median number of individuals each household contributed to the study was 2 (interquartile range [IQR] 1–4). In July 2012, follow-up of all children younger than 5 years was discontinued and 250 additional participants aged 18 years or older were recruited from areas with high malaria transmission in the same village. Every four weeks, study subjects were examined by a clinician and a blood smear was prepared. Participants were also encouraged to go to the study clinic if they developed symptoms. On average, individuals had 11 scheduled and 1.7 unscheduled visits. For detection and quantification of gametocytes, 1,000 white blood cells were counted and smears were read twice for the majority of scheduled visits (5454/5463). An individual was considered gametocytemic if sexual stage parasites were observed in both smear reads or if gametocyte presence was confirmed in a third read whenever only one of the first two reads detected gametocytes. Non-severe clinical malaria episodes were treated with artemether-lumefantrine; severe episodes were treated with quinine or injectable artesunate.

### Ethics

The study protocol and consent were approved by the Institutional Review Board at the National Institute of Allergy and Infectious Diseases, National Institutes of Health, and by the Ethics Committee of the Faculty of Medicine, Pharmacy and Dentistry, University of Bamako. Written informed consent was obtained from study participants or from parents or guardians of children. All procedures were carried out in accordance with the relevant guidelines.

### Mosquito skin feeding assays

Malaria parasite transmission potential of study participants was assessed by mosquito skin feeding experiments, the most sensitive method to quantify infectivity^17^: 95 assays were performed in the first year of the study (June 2011–June 2012), and 44 in the second year (July 2012–June 2013). In each experiment, 60 female *Anopheles gambiae* mosquitoes, distributed in two cups, were allowed to blood-feed directly on the skin of volunteers. Fed mosquitoes were kept for one week before dissection and oocyst detection by microscopy. Only assays with at least 10 dissected mosquitoes were included in this analysis.

### Hemoglobin variants

Hemoglobin variants were ascertained using high-performance liquid chromatography (D-10 instrument; Bio-Rad, Hercules, California, USA): 380 (76.3%), 54 (10.8%), 59 (11.8%), 2 (0.4%) and 3 (0.6%) participants were AA, AS, AC, CC and SC, respectively. Genotypes were equally distributed among individuals from different age groups (P = 0.57) and enrolled in different years (P = 0.66).

### Statistical analysis

To assess the association between hemoglobin variants and parasite and gametocyte carriage, mixed effects logistic models with two random effects (study participant and household) were used. Mixed effects Poisson models for the outcomes of infection and gametocytemia incidences during scheduled visits with one random effect (household) were also fit and similar results were obtained (see Supplementary Information). Multivariate estimates were adjusted for transmission season and age. In analyses excluding infants, who might be more resistant to malaria compared to older children, we obtained similar results for all-age models (data not shown). To quantify the importance of infections in individuals with hemoglobin variants to local malaria epidemiology, we used demographic and parasitological data to calculate the proportions of all patent infections that occur in children and adults with different hemoglobin types (Supplementary Information).

The datasets generated during the current study are available from the corresponding author on reasonable request.

## Results

### Parasite carriage during dry and wet seasons

In Table 1, characteristics of the study population are presented. *P. falciparum* asexual parasites and gametocytes were detected by microscopy at 776/5,457 (14.2%) and 270/5,457 (4.9%) routine visits, respectively. During the transmission seasons (July–December) of 2011 and 2012, 47.7 and 67.9% of the study population, respectively, carried patent asexual parasites during at least one scheduled visit. The number of parasite carriers during dry seasons (January–June) was lower: approximately one sixth and one third of participants had at least one routine visit with patent parasites during the dry seasons of 2012 and 2013, respectively. In parallel, 17.4 and 26.0% of individuals had microscopically-detectable gametocytes during 2011 and 2012 wet seasons, respectively; during dry season months, only half as many participants developed gametocytemia. In addition to routine visits, there were 859 unscheduled visits: asexual parasites were observed at 293/810 (36.2%) visits and gametocytes at 15/808 (1.9%). 609 clinical malaria episodes were diagnosed and treated, mostly (547/609) during wet seasons; 2 were considered severe according to WHO criteria.

**Table 1:** Description of study population.

		N (%)
**Enrollment year**		
2011		250 (50.0)
2012		250 (50.0)
**Gender**		
*Male*		192 (38.4)
*Female*		308 (61.6)
**Age at enrollment (in years)**		
<*5*		102 (20.4)
*5*–*15*		73 (14.6)
>*15*		325 (65.0)
**Bed net use**		
*Yes*		448 (89.6)
*No*		52 (10.4)
**Number of visits**		
*Scheduled*		5,463 (86.4)
*Unscheduled*		859 (13.6)

### Hemoglobin variants have opposing effects on parasite positivity

**Figure 1:**
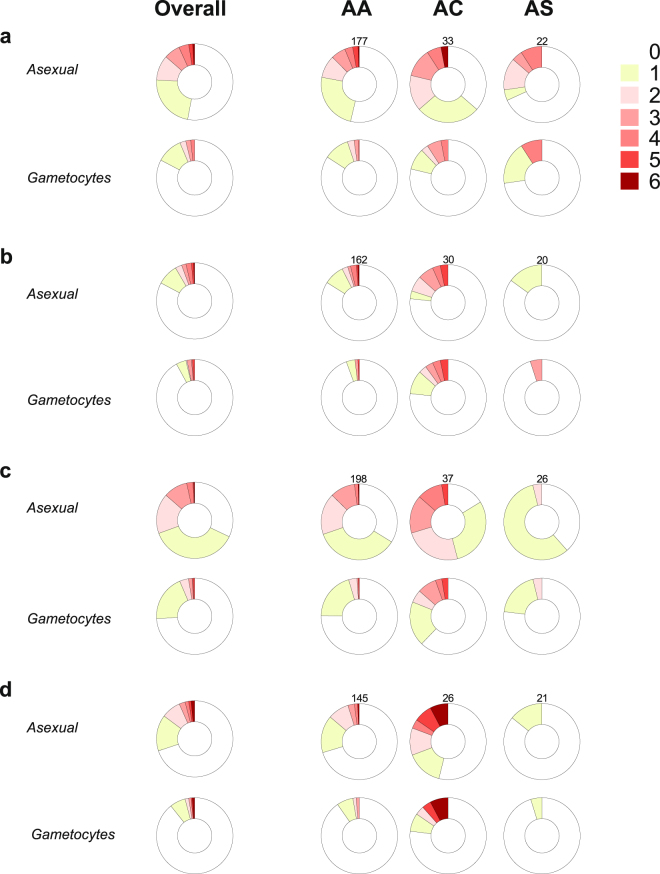
Longitudinal patterns of malaria infection by hemoglobin type. Distribution of study participants by number of scheduled visits with patent parasites during the wet season of the year 2011 (**a**), the dry (**b**) and wet (**c**) seasons of the year 2012 and the dry season (**d**) of the year 2013. Only individuals with 4 or more scheduled visits during each period are included. Numbers above donut charts in each panel indicate the sample sizes of genotype groups for that season.

Asexual stage parasites were more frequently detected in AC versus AA individuals (Fig. 1). Notably, AC and AS genotypes had opposing effects on falciparum infection, defined as the presence of either asexual or sexual stage parasites. In multivariate analyses (Table 2), AC heterozygotes were more often infected than AA individuals (odds ratio [OR] 1.56 95% Confidence Interval [CI] 1.06–2.28, P = 0.02), while sickle cell trait was associated with lower infection risk (OR 0.63 95% CI 0.41–0.97, P = 0.04; reference group, AA genotype). As age influenced parasite carriage and its association with different hemoglobin variants (Supplementary Information Figure S2), age-specific models were developed (Supplementary Information Table S1): only AC children aged 5–15 years had higher risk of patent infection than AA individuals with similar age (OR 3.01 95% CI 1.38–6.57, P = 0.006). Conversely, only adults with AS genotype were less often infected compared to AA individuals with similar age (OR 0.50 95% CI 0.31–0.79, P = 0.003), corroborating previous observations that hemoglobin S-related malaria resistance phenotypes might vary with age^18^. Individuals with AC genotype also had higher asexual stage parasite counts during asymptomatic scheduled visits (median [IQR] asexual stage parasite levels were 43.5 [14.0–127.5] and 22 [4–68] parasites per 1,000 white blood cells [WBCs] in AC versus AA individuals, respectively; P = 0.02 based on mixed effects linear model), which could indicate tolerance to higher parasite densities for longer periods. However, during clinical malaria episodes, when asexual stage parasite counts were on average much higher (median [IQR] densities during clinical malaria and asymptomatic visits were 1,051 [49–5,423] and 26 [5–83] parasites per 1,000 WBCs, respectively), hemoglobin C mutation was not significantly associated with higher asexual stage parasites levels (P = 0.17, Supplementary Information Table S2).

**Table 2:** Mixed effects logistic regression on parasite positivity.

		Odds ratio (95% CI)	P-value
**Hemoglobin type**			
*AA*		—	—
*AC*		1.56 (1.06–2.28)	0.02
*AS*		0.63 (0.41–0.97)	0.04
**Transmission season**			
*Low*		—	—
*High*		4.86 (4.10–5.78)	<0.001
**Age at visit**			
*<5 years*		—	—
*5–15 years*		3.35 (2.34–4.78)	<0.001
*>15 years*		2.26 (1.63–3.14)	<0.001

All visits (scheduled and unscheduled) with microscopy results available were included in this analysis; when only scheduled visits are included, similar results are obtained.

### Children heterozygous for hemoglobin C often carry gametocytes in consecutive study visits

**Figure 2:**
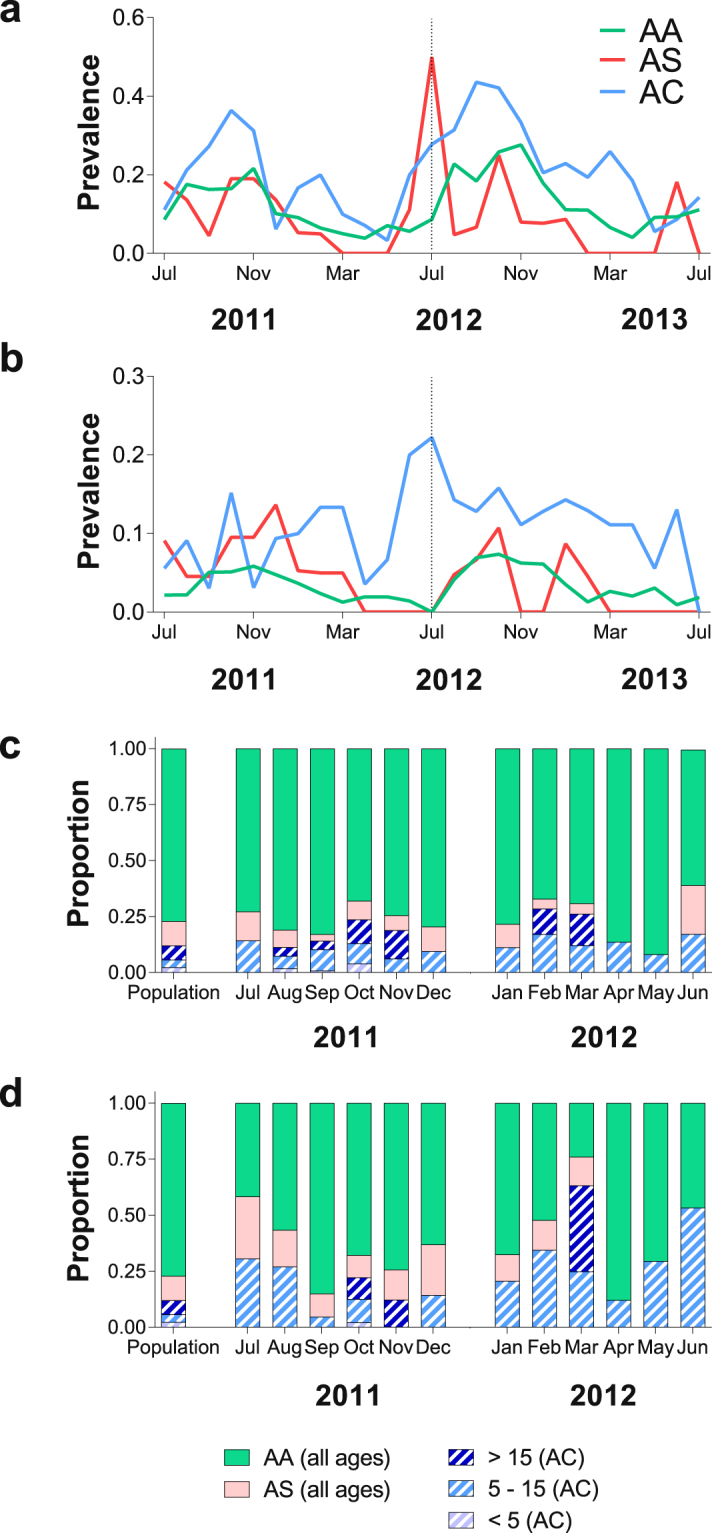
Contributions to the malaria reservoir. Prevalences of (**a**) asexual parasites and (**b**) gametocytes by month and hemoglobin types. The vertical dotted line represents the time-point when 250 additional adults were recruited, and children aged 5 years or less stopped being followed. (**c**,**d**) Proportion of parasitemic individuals in the community from different age groups and with different hemoglobin variants ((**c**) corresponds to infections [asexual or sexual stage parasites]; (**d**), to patent gametocytes); hatched areas represent AC individuals.

**Figure 3:**
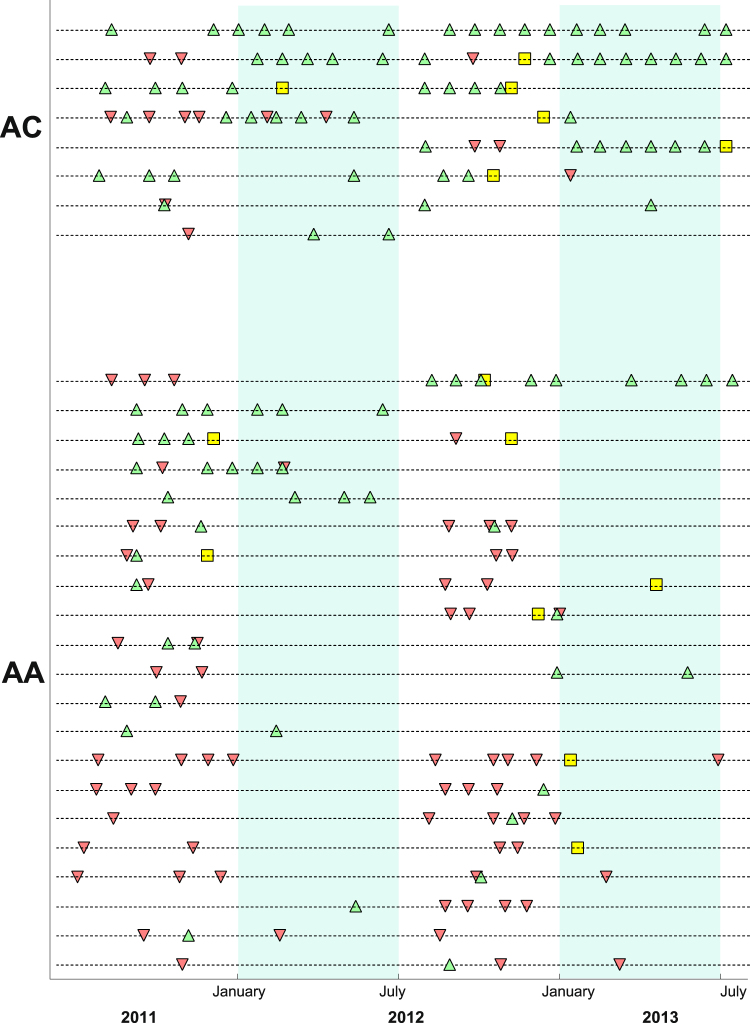
Long-term carriage of patent gametocytes and its temporal relationship with clinical malaria events in school-aged children. Scheduled visits with microscopically-detectable gametocytes are presented as green triangles; inverted red triangles correspond to clinical malaria episodes. Yellow squares represent clinical episodes with patent gametocytes on the day of diagnosis. Each dashed line corresponds to a study participant’s follow-up information. Only AA and AC children aged 5–15 years who carried patent gametocytes at least once during the study were included in this graph. Dry seasons are represented by light green areas. A version of this figure that also includes data from AS schoolchildren and data from children who only developed clinical malaria is presented in the Supplementary Information (Figure S3).

Hemoglobin C heterozygotes also carried patent gametocytes more often than AA and AS individuals (Figs 1 and 2). Age-specific analyses (Supplementary Information Table S3) revealed that AC genotype was associated with higher gametocyte positivity only in school-aged children (OR 5.66 95% CI 1.28–24.96, P = 0.02; reference group, AA school-aged children). The greater probability of detecting falciparum parasites, in particular gametocytes, in AC compared to AA individuals could be linked to chronicity of infections: 6/16 (37.5%) AC children aged 5–15 years had at least three consecutive scheduled visits with patent gametocytes compared to 3/61 (4.9%) AA children of similar age. As expected, long-term carriage of microscopically-detectable levels of gametocytes was often terminated by antimalarials (Fig. 3).

Additional evidence that hemoglobin C might be associated with longer falciparum infections comes from analysis of dry season visits. Parasite carriage throughout dry seasons presumably is explained by long duration infections acquired during previous wet seasons rather than by incident infections. The prevalence of parasitemia among all participants, for example, decreased from 11.2% in January 2012 to 5.4% in April 2012. Between January and June, school-aged children with hemoglobin C mutation were parasitemic more frequently during scheduled visits (OR 5.34 95% CI 0.95–29.95, P = 0.06) than AA children with similar age. At the population level, this group was also a major reservoir of malaria infection (Fig. 2c,d and Supplementary Information): in an analysis accounting for demographic structure and genotype frequencies, AC children aged 5–15 years, who constituted less than 5% of the population, represented 10–15% of patent parasite carriers during dry season months and 10–30% of individuals with microscopically-detectable gametocytes, suggesting that parasites infecting these children might contribute disproportionately to sustaining transmission between wet seasons.

### Frequent gametocytemia in AC children influences transmission potential

Mosquito skin feeding assays were performed throughout the study to directly assess infectiousness of parasitized study participants without knowledge of their hemoglobin type (Supplementary Information Table S4). In the first year of the study, gametocyte-positive participants were recruited for feeding experiments and three age-matched non carriers were selected as controls for each gametocytemic individual: 5/10 (50.0%) experiments involving school-aged AC children resulted in mosquito infections (median [range] percentage of mosquitoes infected 14.6 [12.5–74.0]%), compared to 1/22 (4.5%) assay with an AA child (1/33 mosquito infected); similar results are obtained when only first assays of children participating in multiple experiments are included (2/5 [40.0%] and 1/13 [7.7%], respectively). The median oocyst count in mosquitoes infected by AC schoolchildren was 4 (IQR 1–9, range 1–38), while the sole mosquito infected by an AA school-aged child had one oocyst. These results could be at least partially explained by gametocyte levels of children included in these assays as patent sexual stage parasites were more frequently detected in the AC (7/9) compared to the AA (3/22) group. In the second year of the study, only individuals with patent gametocytes were selected to participate in skin feeding assays: school-aged AC children infected mosquitoes in 2/6 [33.3%] experiments, versus 1/3 [33.3%] for AA children.

## Discussion

Since Allison’s observation that individuals with sickle cell trait were protected against microscopically-detectable infection after parasite exposure or inoculation^19^, our understanding of the interaction between hemoglobin variants and malaria parasites has improved^9,20–28^ but is still incomplete. With the renewed interest in malaria elimination, knowledge on how these prevalent genetic variants influence parasite distribution and cumulative (for example, during an entire dry season) host transmission potential and how malaria transmission processes can vary in communities with different frequencies of these alleles would be particularly valuable. In this longitudinal study, we observed associations between hemoglobin variants and frequencies of patent parasite and gametocyte carriages, possibly linked to differences in infection duration. Our findings provide insights into how these protective genetic factors may affect natural infections and could have practical implications for malaria control and elimination programs in areas where these mutations are common, as treating individuals who are more likely to be infected throughout dry seasons is an effective strategy to reduce seasonal peaks of transmission.

Several studies (reviewed in ref.^4^) have shown that both hemoglobin variants C and S protect against severe malaria. Although it is possible that the mechanisms of protection afforded by these variants target pathogenic events that are unique to severe clinical cases, epidemiological evidence that these variants also influence non-severe clinical malaria risk suggests that heterozygosis for hemoglobin C or S influences pathogenic processes common to all falciparum malaria infections. Experimental work also indicates that these mutations affect malaria parasites in several different ways^29^, however we still do not understand the importance of these different mechanisms in natural infections and how within-host parasite dynamics in AA individuals and in AS or AC heterozygotes differ, especially in terms of duration of parasite circulation and production of transmission stages.

This longitudinal study entailed frequent active follow-up, which is essential for the characterization of asymptomatic falciparum carriage, and included individuals of all ages. We observed that the two hemoglobin variants present in our study population might influence the reservoir of infection in different ways: hemoglobin C mutation is associated with frequent patent carriage of parasites, including gametocytes, in children aged 5–15 years; hemoglobin S heterozygosis is associated with reduced frequency of falciparum positivity in adults. While the protective effect of hemoglobin S against falciparum parasitemia has been described^30,31^, only a few prior studies, using designs different to ours, assessed whether individuals with hemoglobin C mutation were more likely to carry gametocytes. In a cross-sectional study from Ghana^26^, Ringelhann and colleagues observed that AC children carried gametocytes more often than AA children; more recently, large parasitological surveys in Burkina Faso^28^ also showed higher prevalence of gametocytes in AC individuals compared to AA individuals. Together, these previous studies and our data provide growing evidence that hemoglobin C is a risk factor for gametocyte carriage in different settings.

Whether the relatively high frequency of patent parasite and gametocyte carriage in AC children could be explained by the same mechanisms that prevent severe and non-severe clinical disease is not clear. Assuming the risk of acquiring new infections is independent of hemoglobin type for individuals with similar age, one mechanism could be invoked to explain our different observations: hemoglobin C is associated with longer falciparum infections, either due to higher parasitemia threshold for pyrexia in children or else to changes in parasite dynamics that delay reaching the pyrogenic threshold. The higher parasite densities during asymptomatic visits in AC versus AA individuals are consistent with differences in pyrogenic threshold, which would influence treatment-seeking behavior and durations of parasite and gametocyte carriages. Several children in our study developed multiple clinical malaria episodes (Figure S3), all treated with antimalarials, and did not have patent gametocytemia during subsequent scheduled visits, providing additional evidence for a link between susceptibility to symptoms during infection and duration of gametocyte carriage. The frequent presence of patent gametocytes in AC children could then be just a consequence of prolonged infections with higher asexual parasite levels.

An alternative explanation for hemoglobin C’s effect on gametocytemia is that hemoglobin variants might directly influence *in vivo* commitment to transmission stage\s. Limited evidence for this comes from *in vitro* work by Trager and colleagues^32^, who observed that falciparum parasites can produce more gametocytes in reticulocyte-rich blood from individuals with anemia, including individuals with sickle cell anemia or homozygous hemoglobin C disease. The hypotheses that hemoglobin C mutation has a direct effect on gametocytogenesis, and that AC individuals have longer malaria infections and tolerate higher asexual parasite densities without symptoms compared to AA individuals, can be tested in epidemiological studies and in controlled human infection studies with intensive follow-up. Asexual stage parasite and gametocyte quantification in every replication cycle will be necessary to estimate asexual-to-sexual commitment rates, with adjustment for complicating factors such as asynchronicity of falciparum infections, the long maturation of falciparum gametocytes^33^, and effects of treatment. Clinical monitoring can determine the parasite densities associated with fever development in individuals with different genotypes.

The presumed effect of hemoglobin C on gametocyte carriage, regardless of whether it is a direct or indirect effect, is likely to influence the transmission potential of individuals carrying this mutation. Indeed, mosquito skin feeding experiments showed that AC children were often infectious to mosquitoes, which is consistent with previous membrane feeding results and with analyses of bloodfed mosquitoes caught in sentinel houses in Burkina Faso^28^. Of note, xenodiagnostic studies that quantified the infectiousness of individuals living in malaria endemic areas regardless of their parasite status suggest that schoolchildren contribute significantly to malaria transmission^34,35^. The observation that in our study area children aged between 5 and 15 years with hemoglobin C mutation are more often gametocytemic and infectious than other children of similar age suggests that where this variant is prevalent the contribution of schoolchildren to local transmission is enhanced. Large xenodiagnostic studies performed in areas where hemoglobin C mutation is present, with sufficient numbers of individuals of different age groups, will allow a more precise estimation of the importance of AC schoolchildren to transmission.

In summary, this study provides evidence that hemoglobin variants that were, and continue to be^36^, selected for the protection they afford against life-threatening malaria might also have a broader impact on local epidemiology by influencing the frequency (or duration) of parasite, including gametocyte, carriage. Mosquito feeding assays that closely mimic *in natura* transmission indicate that these frequent microscopically-detectable gametocyte levels have a direct impact on infectiousness. Although they comprise less than 5% of the population, AC school-aged children contributed about a third of the sexual stage parasite reservoir from May through August, when transmission transitions from low to high intensity. As costs of molecular assays are gradually decreasing, identification of individuals that persistently carry parasites for long duration after infection might accelerate malaria elimination efforts. This may be especially important in areas where programs have effectively controlled malaria, but a persistent reservoir of infection presents a final obstacle to elimination.

## Electronic supplementary material

Supplementary Information
