# Supplementary material for: Hemoglobin variants shape the distribution of malaria parasites in human populations and their transmission potential
Source: Sci Rep. 2017 Oct 27;7:14267. doi: 10.1038/s41598-017-14627-y (PMC5660173; doi:10.1038/s41598-017-14627-y)
Supplement: Supplementary file 1 — Supplementary Information [file 41598_2017_14627_MOESM1_ESM.pdf]

Supplementary Information

Manuscript:

Hemoglobin variants shape the distribution of malaria parasites in human populations and their transmission potential

Authors:

Gonçalves B.P., Sagara I.., Coulibaly M., Wu Y., Assadou M.H., Guindo A., Ellis R.D., Diakite M., Gabriel E., Prevots D.R., Doumbo O.K., Duffy P.E.

Contributions to the reservoir of malaria infections

In **Figure 2**, the relative contributions of individuals with different hemoglobin variants to the reservoir of malaria infection (panels C and D) were estimated as:

$$C_{ijk} = h_i a_j p_{ijk} / \sum_{i=1}^{m} \sum_{j=1}^{n} h_i a_j p_{ijk}$$

where C_{ijk} corresponds to the proportion of infected individuals in the population during month k with hemoglobin variant i and age j; and h_i and a_j represent the proportion of the population with hemoglobin variant i and in the age category j, respectively. Demographic data for Sub-Saharan Africa were obtained (World Population Prospects, United Nations; esa.un.org/wpp/) and used to estimate a_j. Parasite (asexual or sexual stages) prevalence, based on scheduled visits only, during month k in individuals with hemoglobin variant i and in the age category j is represented by p_{ijk}.

Figure S1. Map with location of Bancoumana, Mali. Map was generated using qGIS version 2.12.0 (http://www.qgis.org/en/site/). Country boundaries and river location were obtained from http://www.gadm.org/ and https://data.humdata.org.

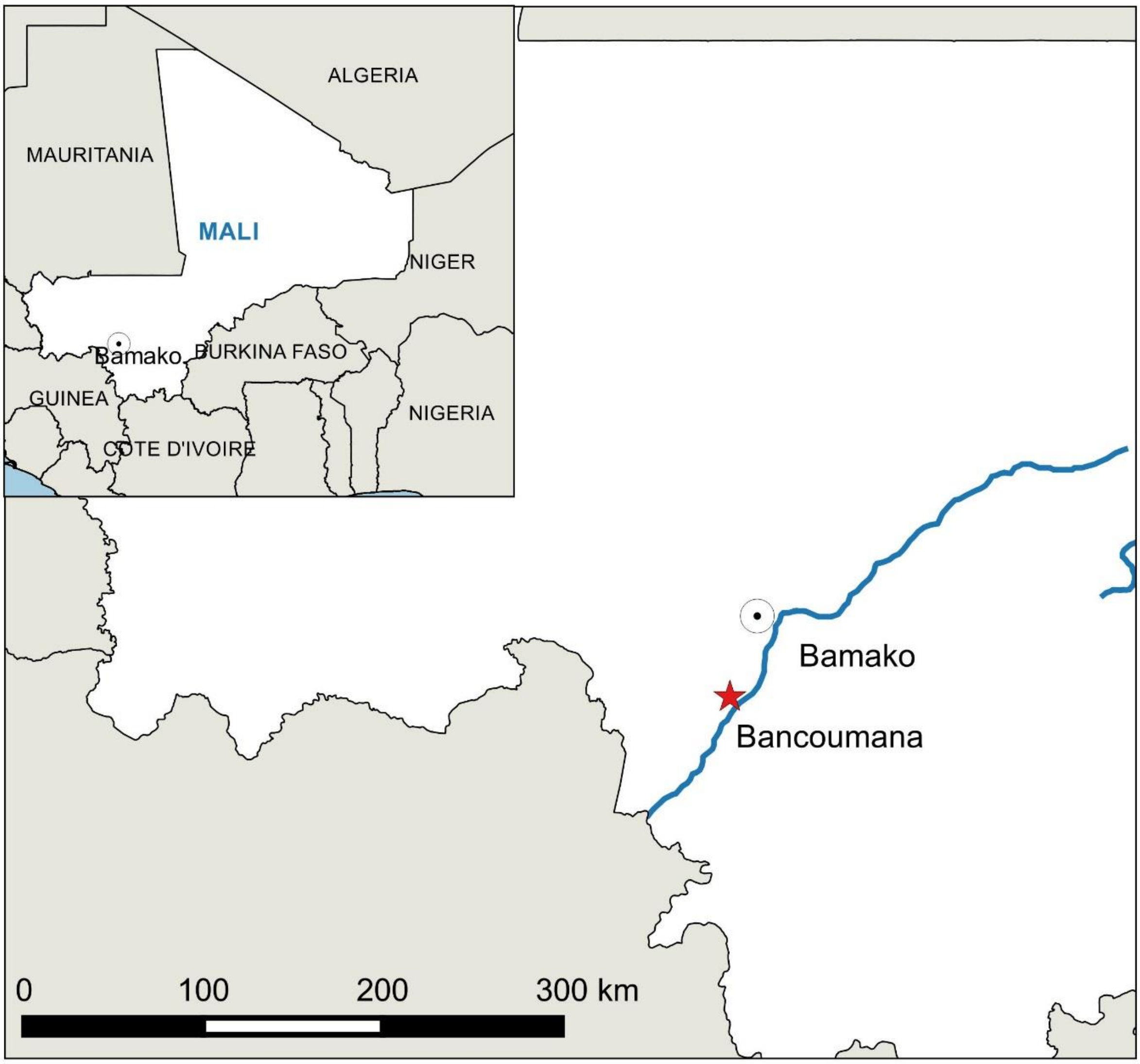

Figure S2. Age-specific prevalences of asexual (A, C, E) and sexual (B, D, F) stage parasites: (A, B) children aged 5 years or less; (C, D) children aged between 5 and 15 years; (E, F) adults. Whenever blood smear results were available for less than five individuals during a particular month (Table S6), estimates were not presented. Of note, children aged 5 years or less were not followed after June 2012.

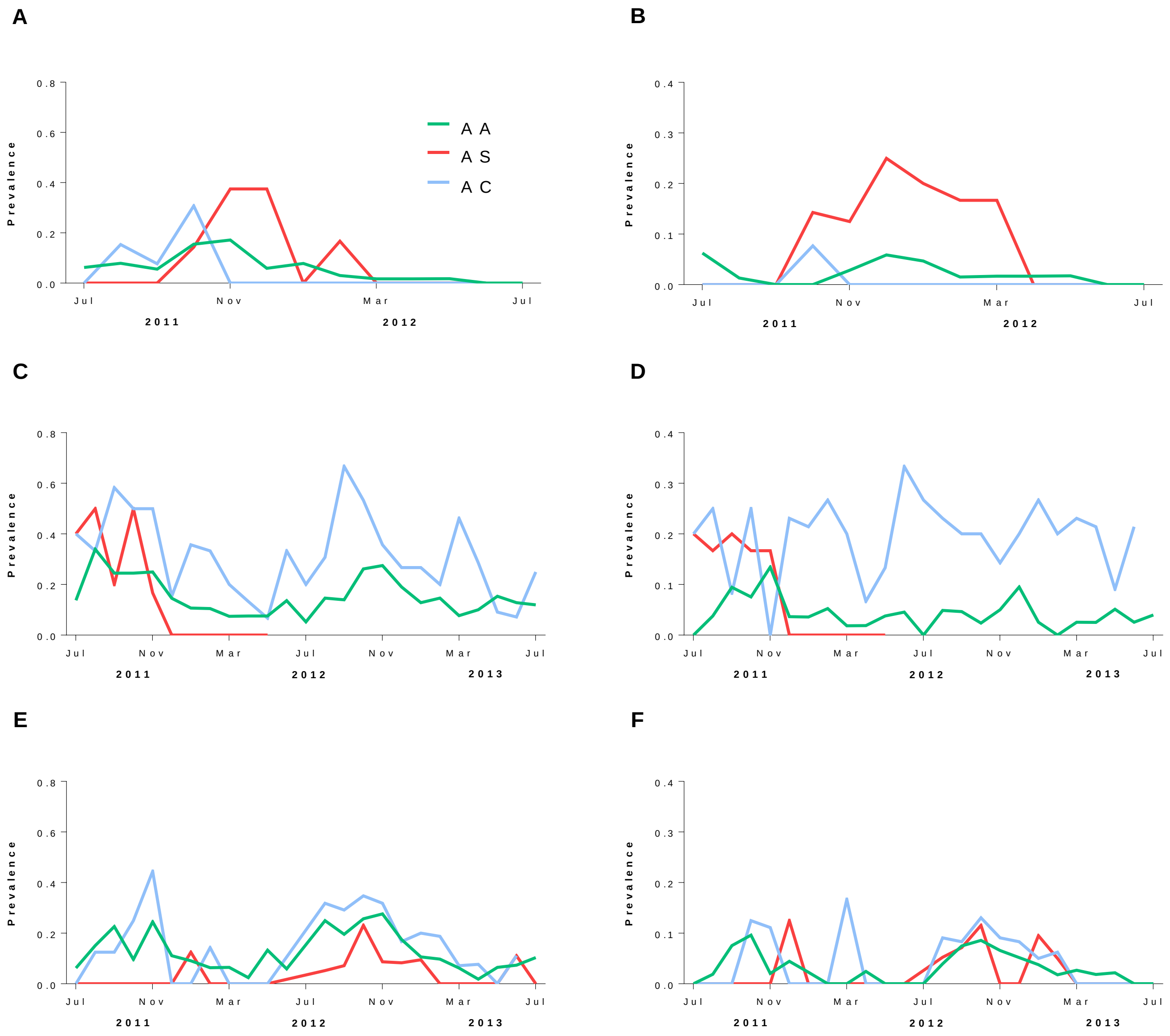

Figure S3. Long-term carriage of patent gametocytes and its temporal relationship with clinical malaria events in school-aged children. Scheduled visits with microscopically-detectable gametocytes are presented as green triangles; inverted red triangles correspond to clinical malaria episodes. Yellow squares represent clinical episodes with patent gametocytes on the day of diagnosis. Each line and space between consecutive lines correspond to a study participant's follow-up information. Only children aged 5 – 15 years who carried patent gametocytes at least once during the study or who developed clinical malaria were included in this graph. Dry seasons are represented by light green areas.

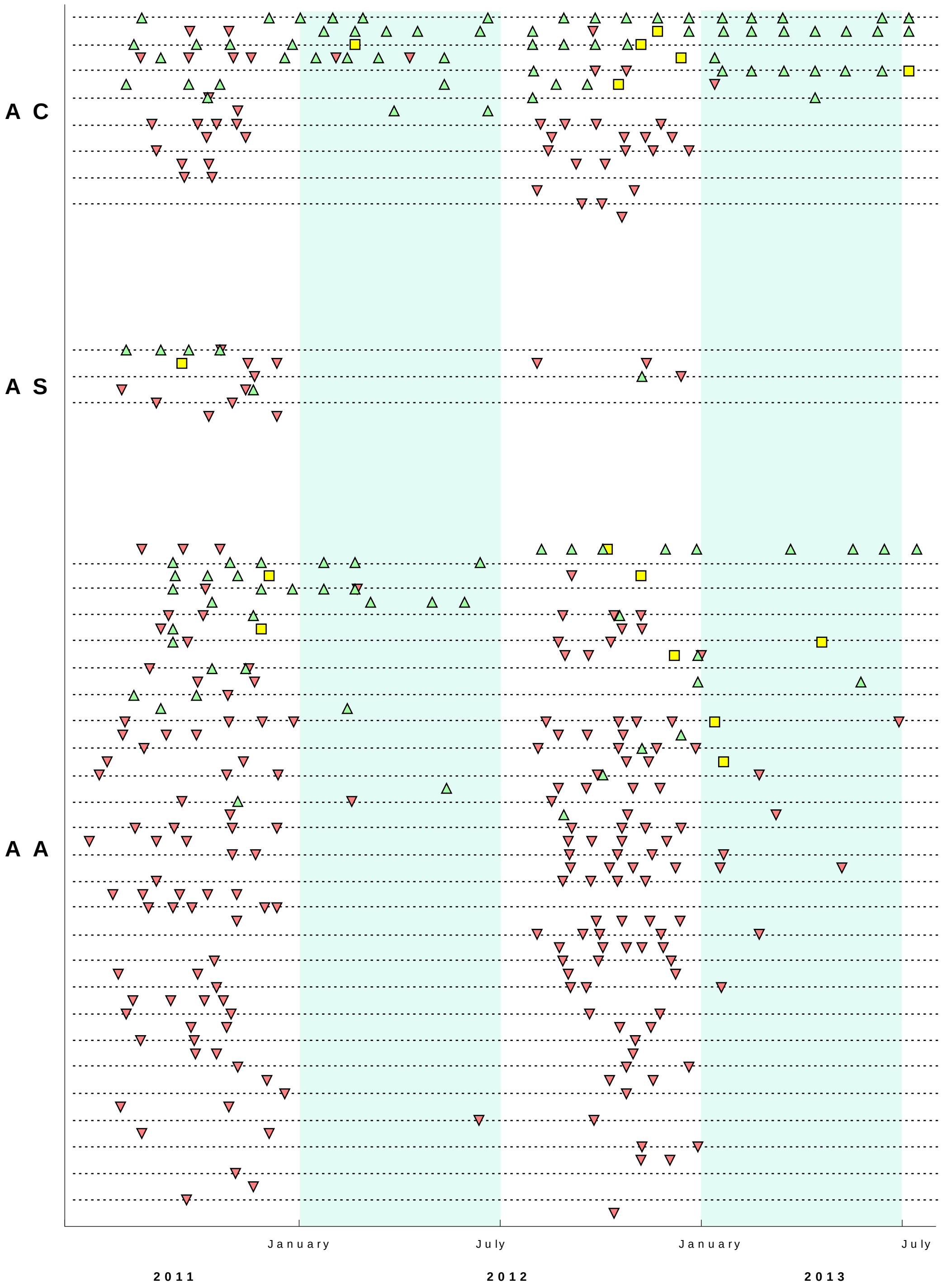

Table S1. Age-specific mixed effects logistic models on parasite positivity by microscopy. Results are adjusted for transmission season. Both scheduled and unscheduled visits were included in these analyses.

**Children aged < 5 years**		**Odds ratio (95% CI)**	**P-value**
**Hemoglobin type**			
	AA	-	-
	AC	0.76 (0.22 – 2.60)	0.66
	AS	1.22 (0.29 – 5.15)	0.78

**Children aged 5-15 years**		**Odds ratio (95% CI)**	**P-value**
**Hemoglobin type**			
	AA	-	-
	AC	3.01 (1.38 – 6.57)	0.006
	AS	1.02 (0.32 – 3.26)	0.98

**Individuals aged > 15 years**		**Odds ratio (95% CI)**	**P-value**
**Hemoglobin type**			
	AA	-	-
	AC	1.39 (0.91 – 2.13)	0.12
	AS	0.50 (0.31 – 0.79)	0.003

Table S2. Mixed effects linear regression on log-transformed asexual parasite levels. Only visits with microscopically-detectable asexual falciparum parasites were included in this analysis. (A) Only scheduled visits without evidence of malaria symptoms were included (routine visits occurring 3 days or less after the onset of clinical symptoms were considered symptomatic); (B) Only scheduled or unscheduled visits classified as clinical malaria by study clinician were included.

(A)

		Coefficients (95% CI)	P-value
**Hemoglobin type**			
	AA	-	-
	AC	0.25 (0.04 – 0.46)	0.02
	AS	-0.05 (-0.37 – 0.28)	0.78
**Transmission season**			
	Low	-	-
	High	0.34 (0.20 – 0.48)	<0.001
**Age at visit**			
	< 5 years	-	-
	5 – 15 years	0.36 (0.07 – 0.64)	0.01
	> 15 years	-0.02 (-0.29 – 0.25)	0.88

(B)

		Coefficients (95% CI)	P-value
**Hemoglobin type**			
	AA	-	-
	AC	-0.20 (-0.48 – 0.08)	0.17
	AS	-0.27 (-0.65 – 0.11)	0.17
**Transmission season**			
	Low	-	-
	High	1.04 (0.66 – 1.42)	<0.001
**Age at visit**			
	< 5 years	-	-
	5 – 15 years	-0.06 (-0.34 – 0.22)	0.68
	> 15 years	-1.17 (-1.45 – -0.89)	<0.001

Table S3. Age-specific mixed effects logistic models on gametocyte positivity. All visits (scheduled and unscheduled) with microscopy results available were included in this analysis; when only scheduled visits are included, similar results are obtained.

**Children aged < 5 years**			
		**Odds ratio (95% CI)**	**P-value**
**Hemoglobin type**			
	AA	-	-
	AC	0.47 (0.02 – 11.06)	0.64
	AS	10.94 (0.83 – 144.25)	0.07
**Children aged 5-15 years**			
		**Odds ratio (95% CI)**	**P-value**
**Hemoglobin type**			
	AA	-	-
	AC	5.66 (1.28 – 24.96)	0.02
	AS	3.49 (0.40 – 30.51)	0.26
**Individuals aged > 15 years**			
		**Odds ratio (95% CI)**	**P-value**
**Hemoglobin type**			
	AA	-	-
	AC	1.66 (0.82 – 3.39)	0.16
	AS	0.83 (0.38 – 1.85)	0.66

Table S4. Mosquito skin feeding experiments.

		First year	*Second year*
**Number of assays**		95	44
		**N (%)**	
**Age (in years)**			
	< 5	10 (10.5)	-
	5 – 15	40 (42.1)	10 (22.7)
	> 15	45 (47.4)	34 (77.3)
**Hemoglobin type**			
	AA	67 (70.5)	30 (68.2)
	AC	20 (21.0)	10 (22.7)
	AS	7 (7.4)	4 (9.1)
	SC	1 (1.0)	-
**Gametocyte positive**		27 (29.3)	43 (97.7)[#]
**Positive feeding assays***		13 (17.8)	17 (38.6)
		**Median (IQR)**	
**Mosquitoes dissected per assay**		32 (20 - 42)	42 (38 - 46)
**% of infected mosquitoes in positive assays**		13.3 (9.1 - 22.9)	17.8 (10.0 - 62.9)

*Positive assays were those with at least one infected mosquito; only assays with at least 10 dissected mosquitoes are considered

[#]One individual had gametocytes detected only in the first of the three smear reads

Table S5. Mixed effects Poisson models on infection and gametocyte positivity. In these models, the number of scheduled visits each individual had during the follow-up was used as an exposure variable. (A) Model on infection incidence during scheduled visits; (B) Model on gametocyte incidence during scheduled visits (the association between AC genotype and gametocyte incidence was also observed when using a mixed effects negative binomial model with robust standard errors [data not shown]).

(A)

		Incidence rate ratio (95% CI)	P-value
**Hemoglobin type**			
	AA	-	-
	AC	1.46 (1.16 – 1.83)	0.001
	AS	0.68 (0.48 – 0.96)	0.03
**Age at enrollment**			
	< 5 years	-	-
	5 – 15 years	2.75 (2.13 – 3.55)	<0.001
	> 15 years	1.89 (1.48 – 2.40)	<0.001

(B)

		Incidence rate ratio (95% CI)	P-value
**Hemoglobin type**			
	AA	-	-
	AC	2.80 (1.80 – 4.35)	<0.001
	AS	1.31 (0.71 – 2.42)	0.39
**Age at enrollment**			
	< 5 years	-	-
	5 – 15 years	2.33 (1.48 – 3.67)	<0.001
	> 15 years	1.29 (0.83 – 2.01)	0.25

Table S6. Number of study participants with blood smear results available during (A) the wet season of the year 2011 and (B) the dry season of the year 2012.

(A)

	< 5 years			5 - 15 years			> 15 years		
	AA	*AC*	*AS*	*AA*	*AC*	*AS*	*AA*	*AC*	*AS*
**July**	32	6	1	29	5	5	32	7	5
**August**	76	13	8	53	12	6	53	8	8
**September**	72	13	8	53	12	5	53	8	9
**October**	71	13	7	53	12	6	52	8	8
**November**	70	11	8	52	12	6	49	9	7
**December**	68	11	8	55	13	6	45	8	8

(B)

	< 5 years			5 - 15 years			> 15 years		
	AA	*AC*	*AS*	*AA*	*AC*	*AS*	*AA*	*AC*	*AS*
**January**	64	9	5	56	14	6	44	7	8
**February**	66	8	6	57	15	6	47	7	8
**March**	59	9	6	54	15	6	46	6	8
**April**	60	8	5	53	15	6	41	5	8
**May**	57	9	6	53	15	6	45	6	7
**June**	32	6	5	22	9	1	17	0	3
